# Supplementary material for: Physiological basis for atmospheric methane oxidation and methanotrophic growth on air
Source: Nat Commun. 2024 May 16;15:4151. doi: 10.1038/s41467-024-48197-1 (PMC11519548; doi:10.1038/s41467-024-48197-1)
Supplement: Supplementary file 1 — Supplementary information [file 41467_2024_48197_MOESM1_ESM.pdf]

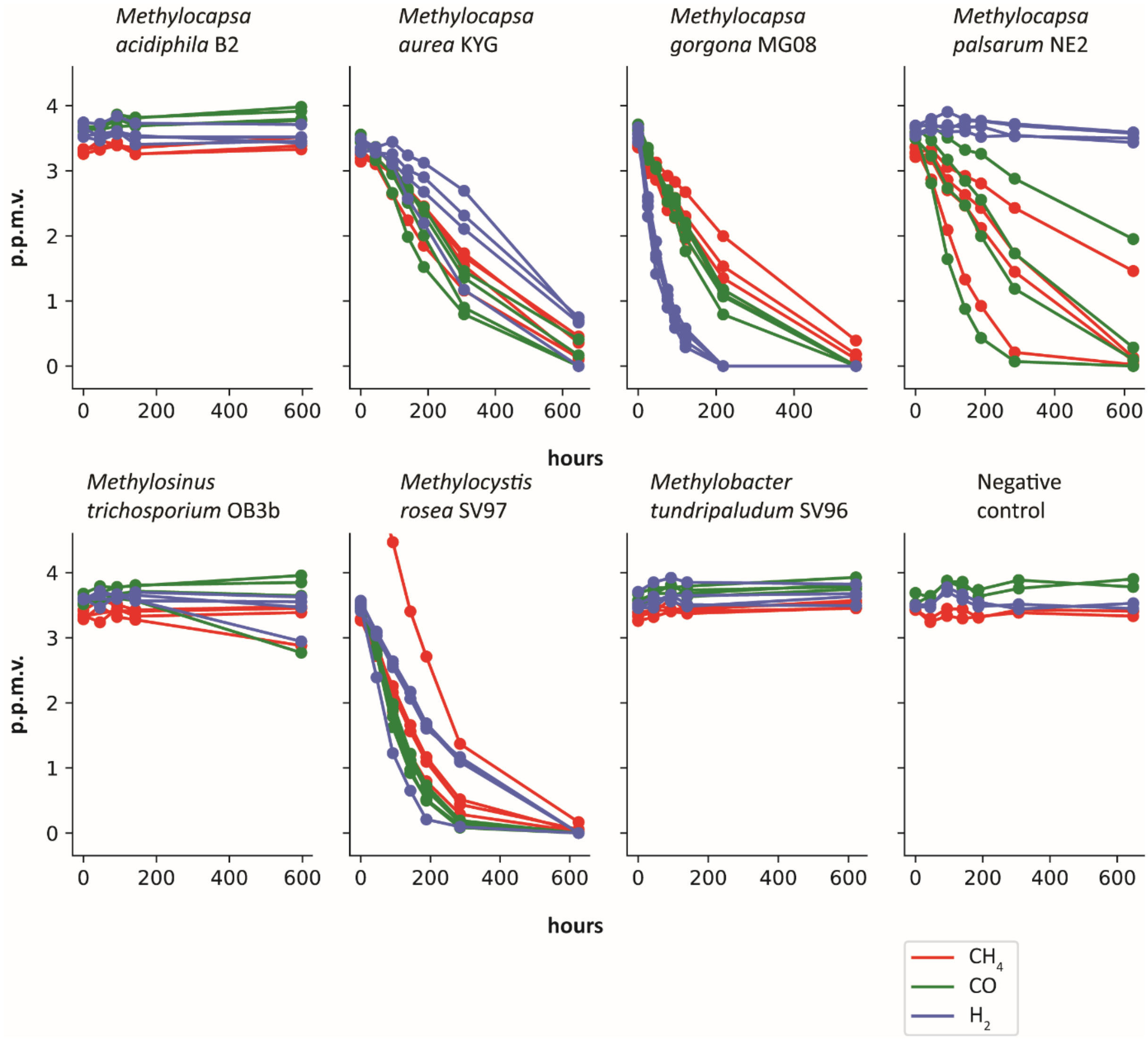

Supplementary Figure 1. Trace gas oxidation on air. CH_4, H_2, and CO oxidation CO displayed in red, blue, and green, respectively, at atmospheric pressure by *M. acidiphila* B2, *M. aurea* KYG, *M. gorgona* MG08, *M. palsarum* NE2, *M. trichosporium* OB3b, *M. rosea* SV97, and *M. tundripaludum* SV96 after 12 months of incubation with air as sole carbon and energy source. Source data are provided in the Source Data file and in the Supplementary Data file (Dataset 1).

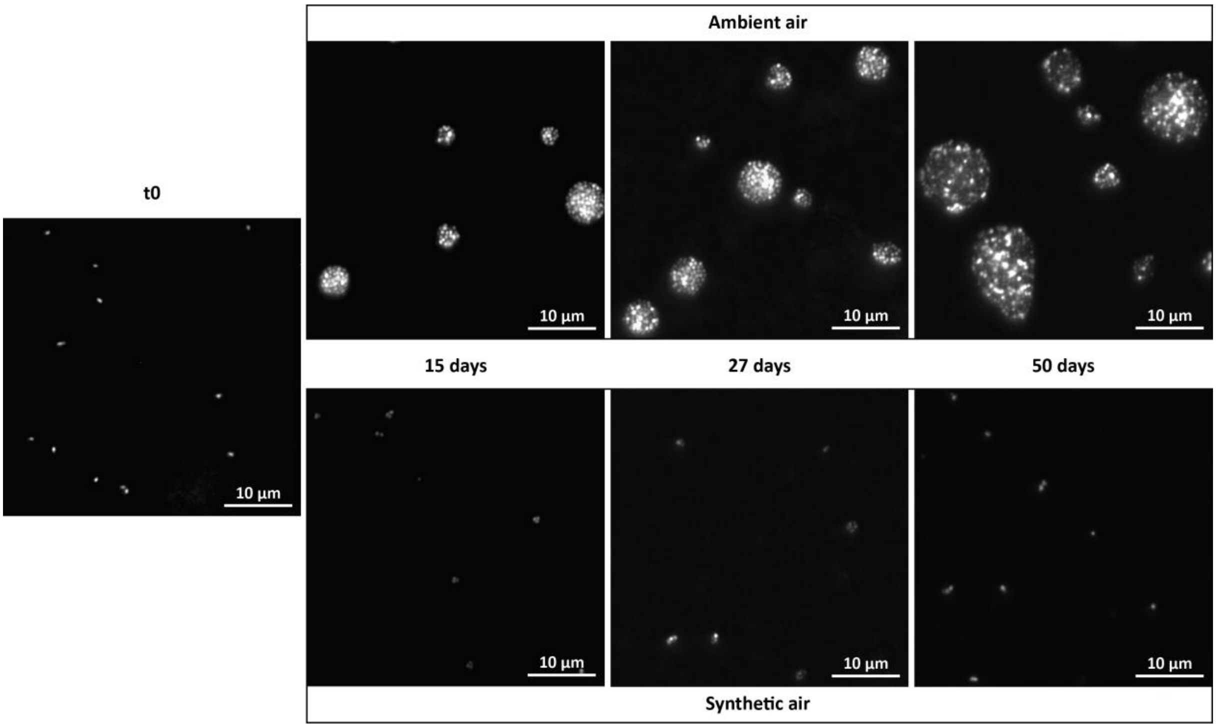

Supplementary Figure 2. Growth control on air with and without trace gases. SYBR green I stained cells of *M. gorgona* MG08 incubated for 15, 27, and 50 days under an atmosphere of ambient air and an atmosphere of synthetic air without the atmospheric trace gases CH_4, CO, and H_2.

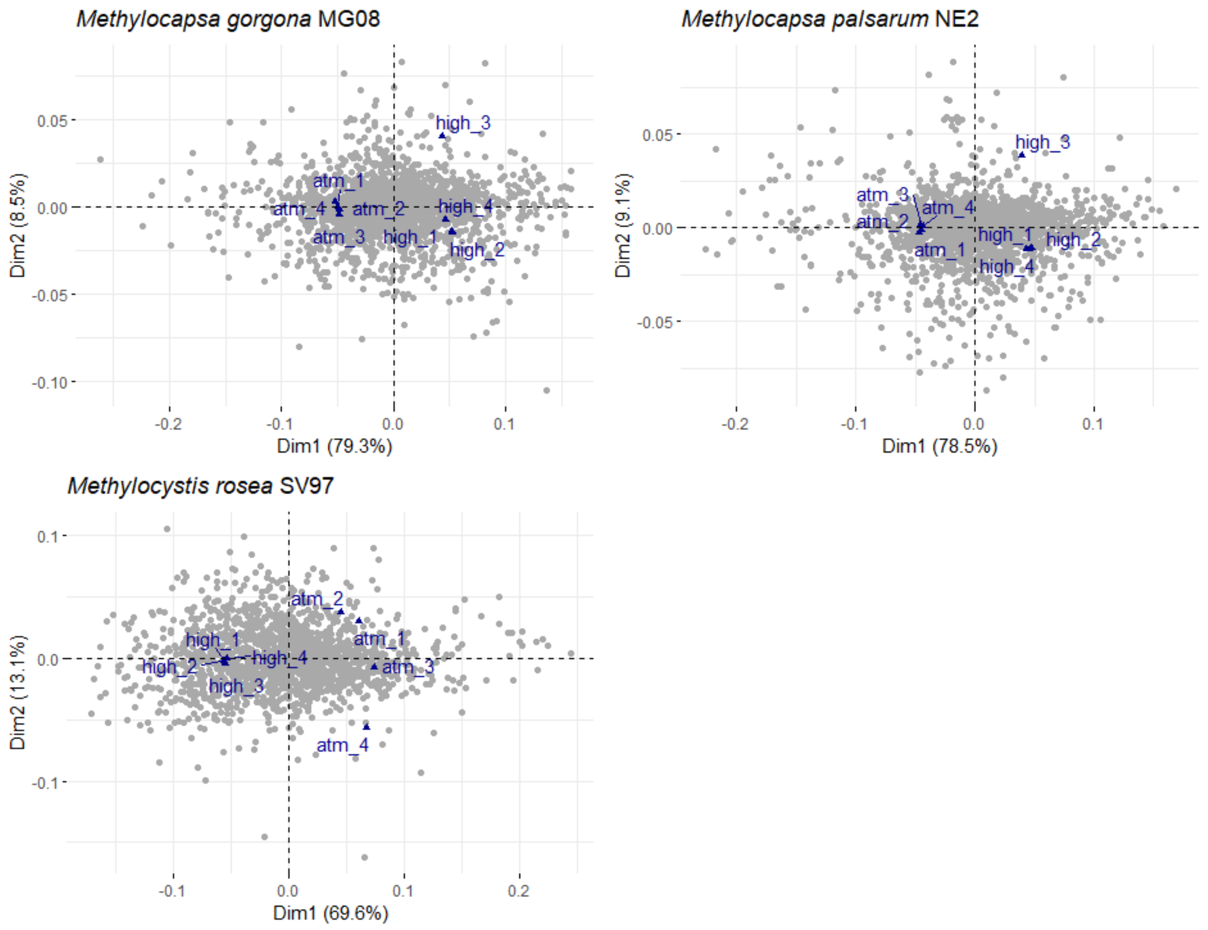

Supplementary Figure 3. CH_4 concentration dependent proteome shifts. Correspondence analysis biplot of protein abundances of *M. gorgona* MG08, *M. palsarum* NE2, and *M. rosea* SV97 at 1.9 p.p.m.v. (atm) and 1000 p.p.m.v. (high) methane in air. Biological replicates (n = 4) incubated at the two CH_4 concentrations are shown in blue. Proteins are shown in grey. Dim – dimension. Source data are provided in the Source Data file and in the Supplementary Data file (Dataset 6 – 8).

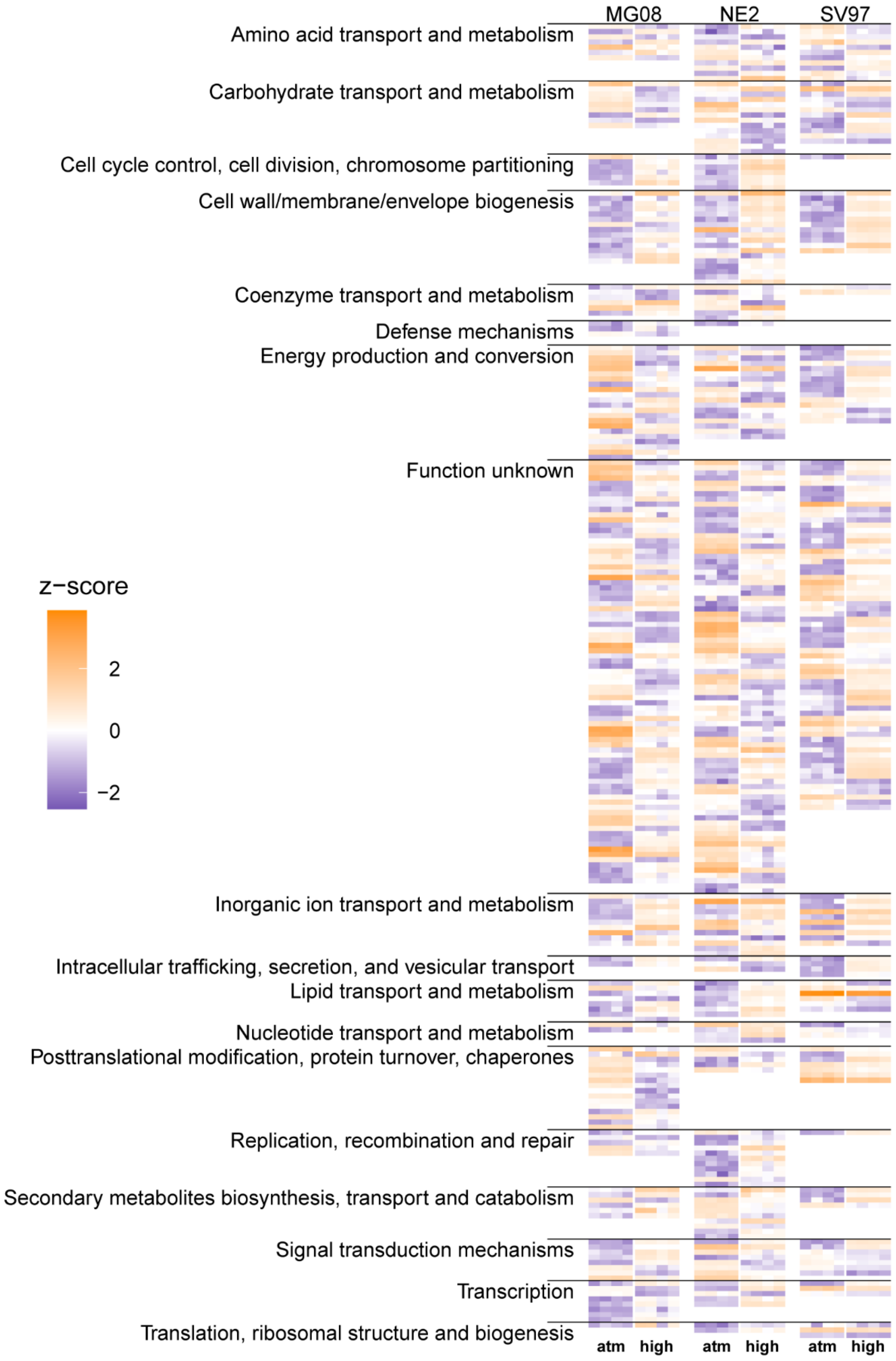

Supplementary Figure 4. CH_4 concentration dependent proteome allocation. Comparative proteomics of *M. gorgona* MG08, *M. rosea* SV97, and *M. palsarum* NE2 exposed to 1000 p.p.m.v. CH_4 (high) in air and 1.9 p.p.m.v. CH_4 (atm) in air. n = 4 biological replicates per CH_4 concentration. Normalized and standardized expression of top 10% proteins contributing most to the inertia of the correspondence analyses (Supplementary Figure 3). High relative abundance = orange, low relative abundance expression = blue. The proteins are grouped by the hierarchical EggNOG categories. Source data are provided in the Source Data file and in the Supplementary Data file (Dataset 12 – 14).

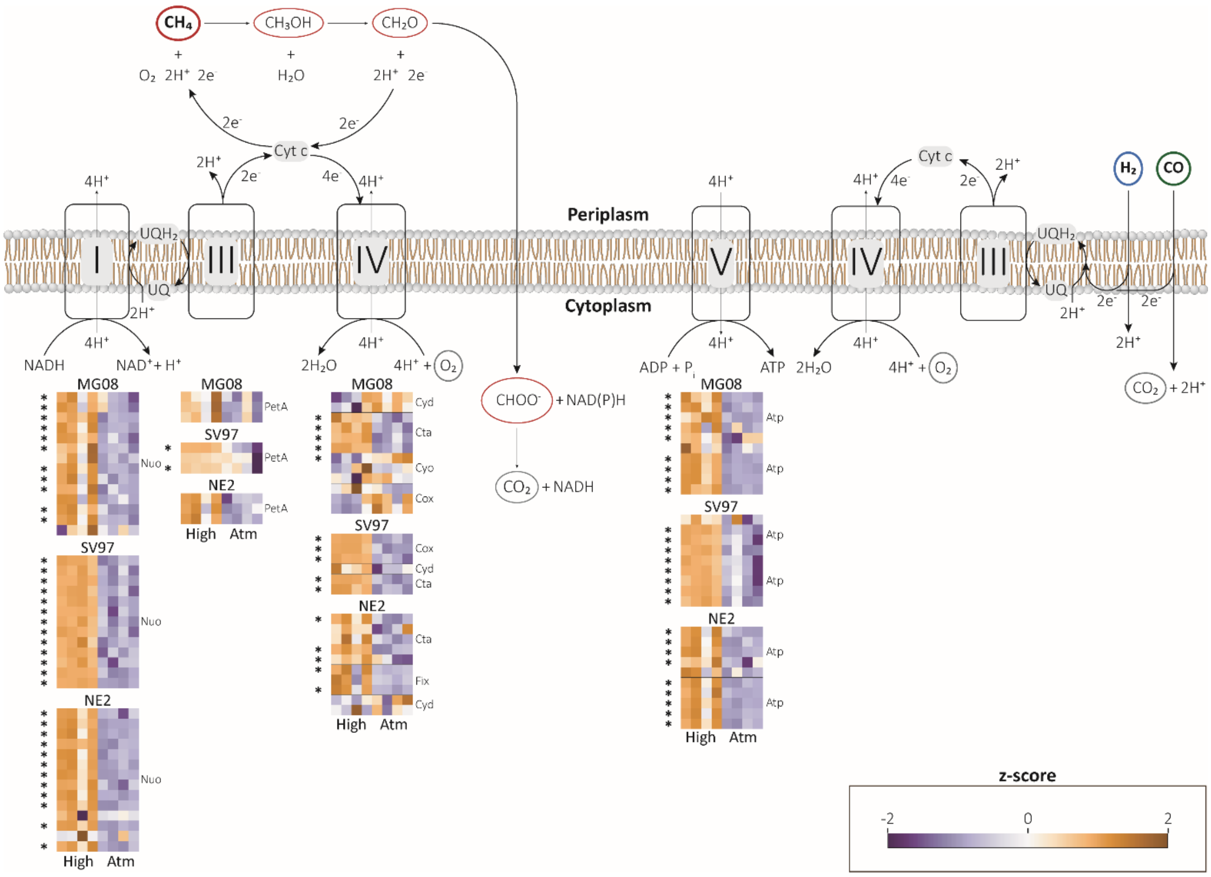

Supplementary Figure 5. Metabolic adjustments during growth on air. Comparative proteomics of *M. gorgona* MG08, *M. rosea* SV97, and *M. palsarum* NE2 exposed to 1000 p.p.m.v. CH_4 (High) in air and 1.9 p.p.m.v. (Atm) CH_4 in air. Normalized and standardized expression of enzymes involved in the electron transport chain. n = 4 biological replicates per CH_4 concentration. High relative abundance = orange, low relative abundance expression = blue. * indicates significant difference in expression between treatments (two sided t-test). Horizontal lines in the heatmaps separate operons of enzymes catalyzing the same reaction. I – NADH ubiquinone oxidoreductase, III – ubiquinol-cytochrome c reductase, IV – cytochrome c oxidase, V – ATP synthase. Source data are provided in the Source Data file and in the Supplementary Data file (Dataset 5).

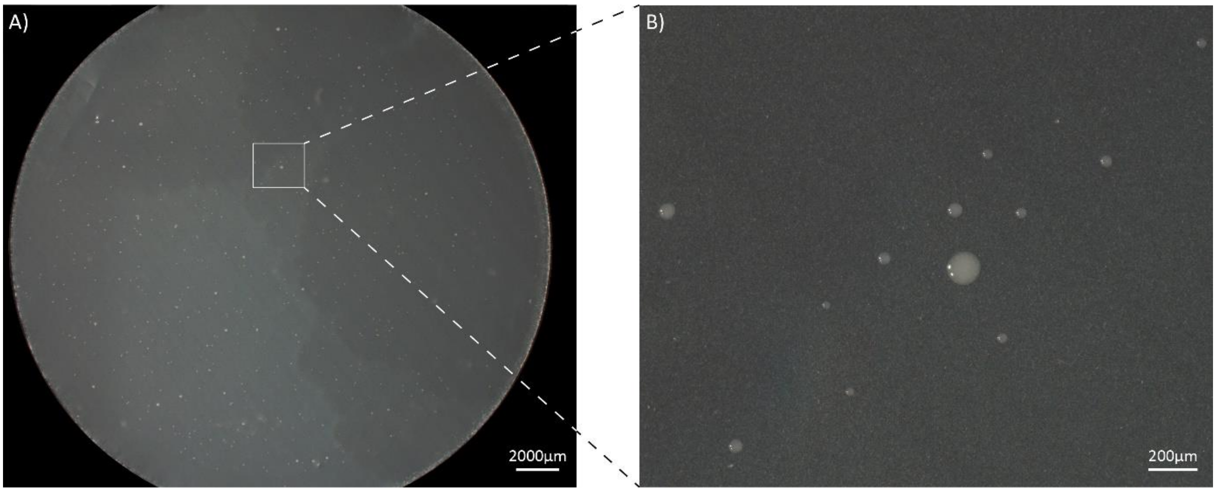

Supplementary Figure 6. Growth with air as nitrogen source. Light microscopic image of a *Methylocapsa gorgona* MG08 culture on a polycarbonate filter after a 12-months incubation period on a carbon- and nitrogen-free growth medium.

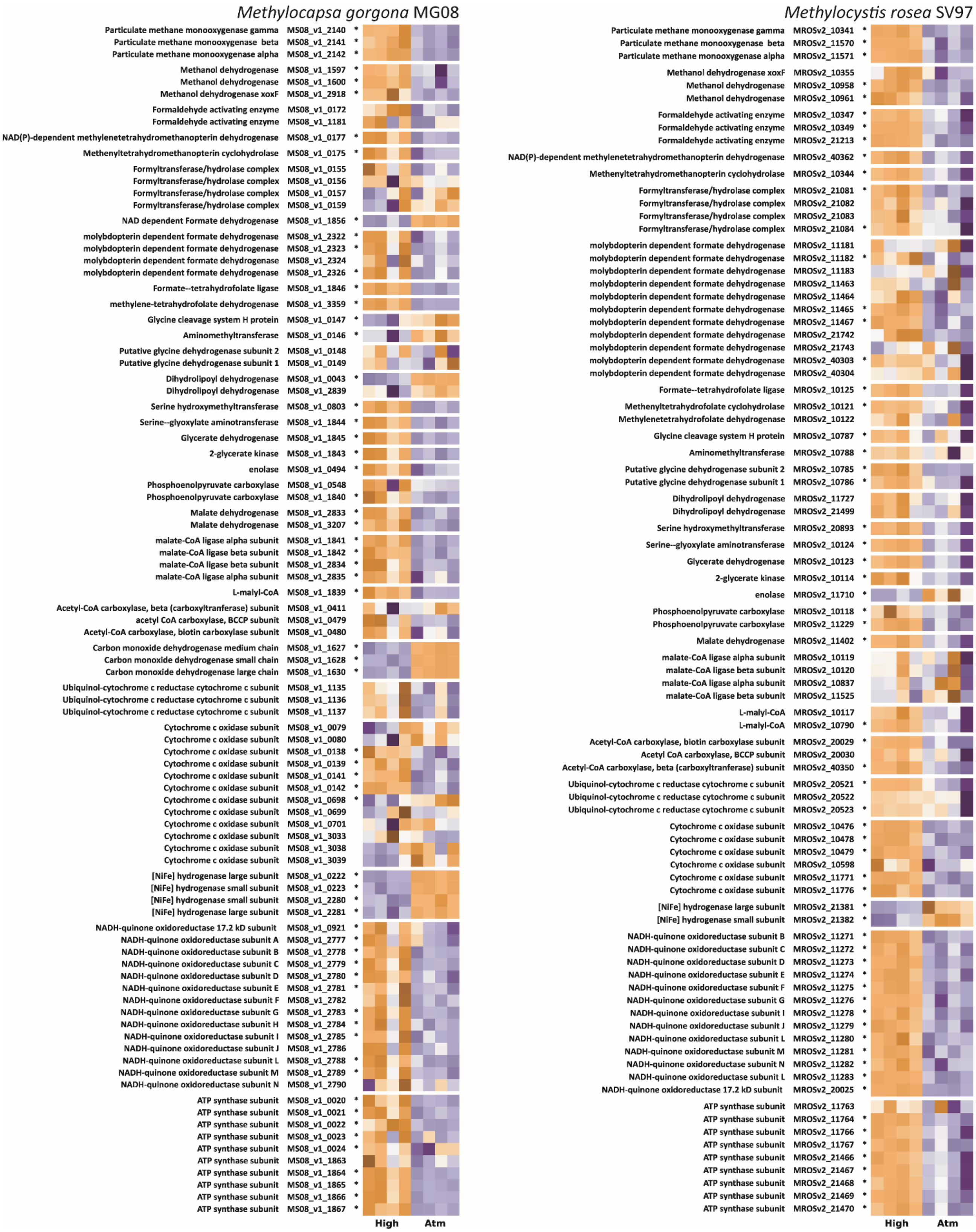

Supplementary Figure 7. Metabolic adjustments during growth on air. Comparative proteomics of *M. gorgona* MG08, *M. rosea* SV97 exposed to 1000 p.p.m.v CH_4 (High) in air and 1.9 p.p.m.v. (Atm) CH_4 in air. Normalized and standardized expression of enzymes involved in the central carbon and energy metabolism is shown. Same heatmaps as in Figure 4 and Supplementary Figure 5 but with protein name and MicroScope accession number. * indicates significant difference in expression between treatments (two sided t-test). Source data are provided in the Source Data file and in the Supplementary Data file (Dataset 5).

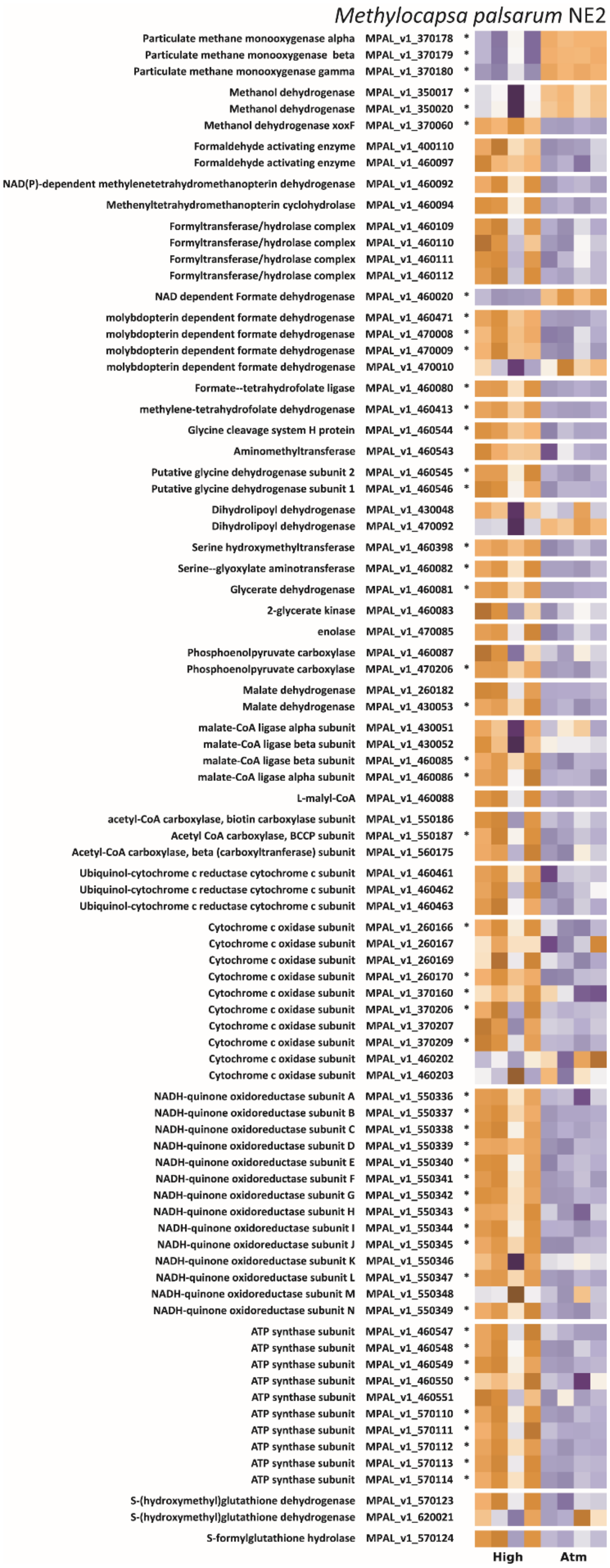

Supplementary Figure 8. Metabolic adjustments during growth on air. Comparative proteomics of *M. palsarum* NE2 exposed to 1000 p.p.m.v. CH_4 (High) in air and 1.9 p.p.m.v. (Atm) CH_4 in air. Normalized and standardized expression of enzymes involved in the central carbon and energy metabolism is shown. Same heatmaps as in Figure 4 and Supplementary Figure 5 but with protein name and MicroScope accession number. *indicates significant difference in expression between treatments (two sided t-test). Source data are provided in the Source Data file and in the Supplementary Data file (Dataset 5).

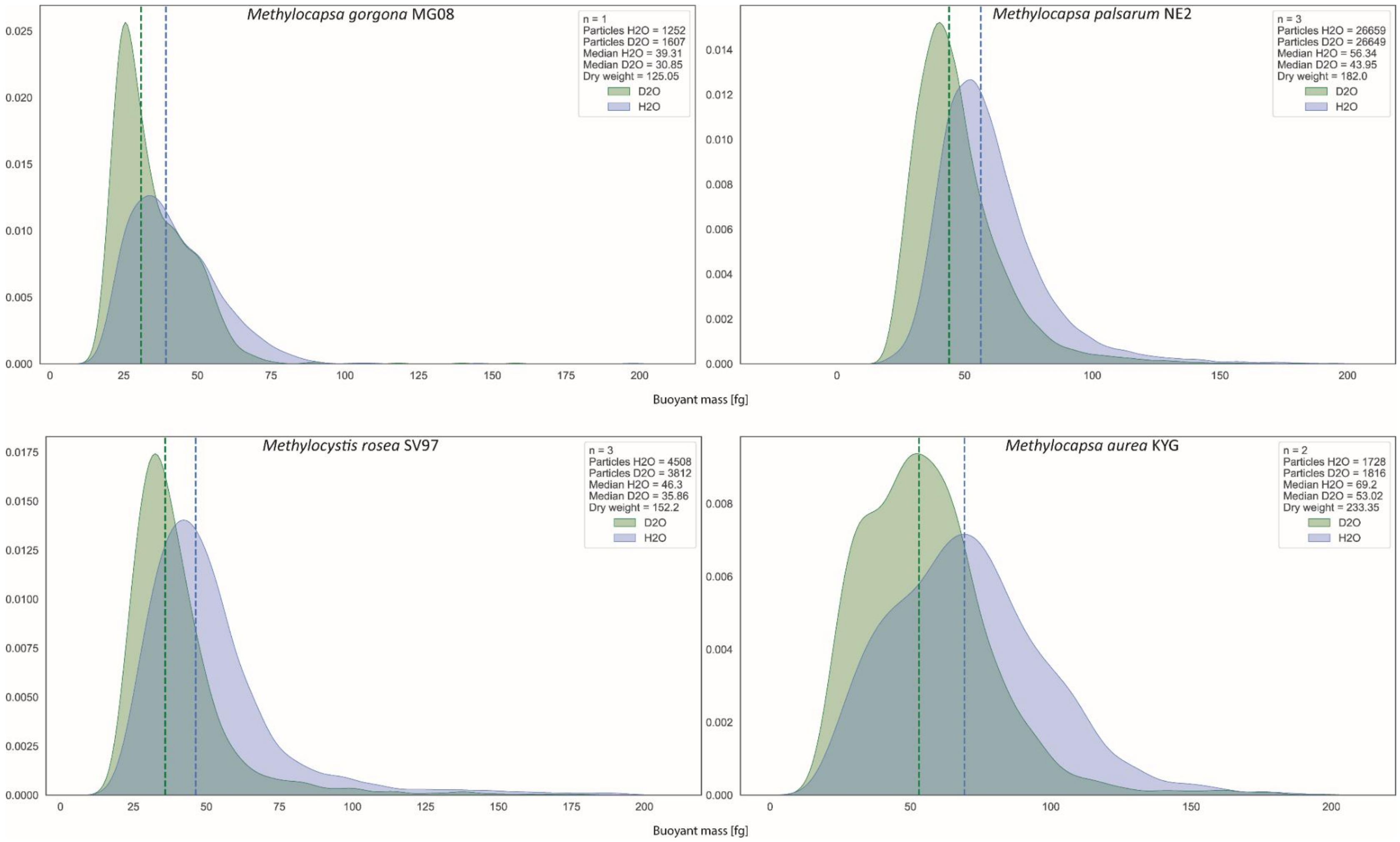

Supplementary Figure 9. Cellular dry weight estimations. Median and buoyant mass distribution of cells of the respective strains in the D_2O-based (green) and H_2O-based (blue) solution. n - number of biological replicates pooled for analysis, Particles H2O - Number of cells measured in H_2O-based solution contributing to the median and distribution, Particles D2O - Number of cells measured in D_2O-based solution contributing to the median and distribution. Median H2O – median of buoyant mass in H_2O-based solution in femtogram, Median D2O – median of buoyant mass in D_2O-based solution in femtogram, Dry weight – estimated cellular dry weight derived from the median of buoyant mass in H_2O-based and D_2O-based solutions in femtogram. Source data are provided in the Source Data file.

BD FACSDiva 8.0.1

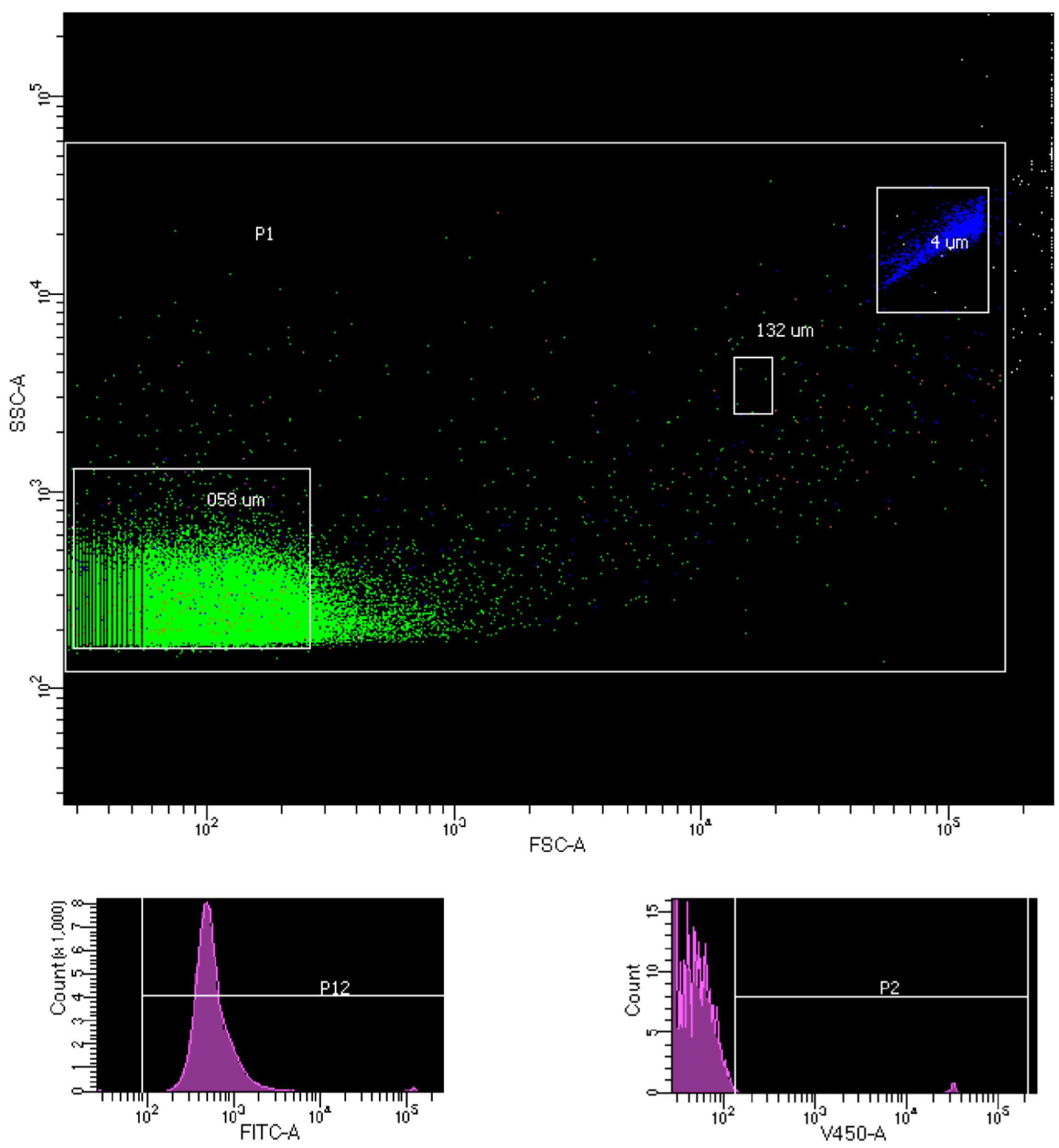

Supplementary Figure 10. Gating strategy for flow cytometry based cell quantification. Flow cytometry gating strategy example for the cell quantification of filter cultures. SYBR green I stained *M. gorgona* MG08 cells in green. 4 µm counting beads in blue. “058 um” – gate defined using 0.58 µm size beads. “132 um” – gate defined using 1.32 µm size beads. “4 um” - gate defined using 4 µm counting beads. P1 – events of interest for the quantification of cells. SSCA-A – side scatter. FSC- A – forward scatter. FITC-A – signal intensity of events detected in the green channel (*M. gorgona* MG08 cells + counting beads). V450-A – signal intensity of events detected in the blue channel (counting beads).
