# Supplementary material for: Physiological basis for atmospheric methane oxidation and methanotrophic growth on air
Source: Nat Commun. 2024 May 16;15:4151. doi: 10.1038/s41467-024-48197-1 (PMC11519548; doi:10.1038/s41467-024-48197-1)
Supplement: Supplementary file 3 — Description of Additional Supplementary Files [file 41467_2024_48197_MOESM3_ESM.pdf]

Description of Additional Supplementary Files:

Supplementary Data 1: Trace gas oxidation: Trace gas oxidation after six to 12 months of incubation with air as sole carbon and energy source

Supplementary Data 2: Oxidation rate: Calculation of cellular oxidation rates of M. aurea KYG, M. gorgona MG08, M. palsarum NE2, and M. rosea SV97 at atmospheric concentrations of CH4, H2, and CO

Supplementary Data 3: Energy calculations: Energy calculation. Average energy yield of M. aurea KYG, M. gorgona MG08, M. palsarum NE2, and M. rosea SV97 per cell from the oxidation of air (Fig. 1B). Average energy yield of M. aurea KYG, M. gorgona MG08, M. palsarum NE2, and M. rosea SV97 per C-mol from the oxidation of air (Fig. 3).

Supplementary Data 4: Gibbs free energy: Calculation of the gibbs free energy change at amtospheric CH4, H2, and CO.

Supplementary Data 5: Comp. proteomics: Proteins involved in trace gas oxidation, carbon assimilation, and the electron transport chain (Fig. 3, Supplementary Fig.4, Supplementary Fig.5-6). Microscope platform annotations and protein abundances used for the comparative proteomics analysis: "trace gas oxidation, carbon assimilation, and the electron transport chain", Label - Gene ID used by Microscope platform, Organism - strain designation used by Microscope platform (Beijerinckiaceae sp. MG08 WGS MS08 = Methylocapsa gorgona MG08), Protein product - protein designation by Microscope platform, Curated name - protein designation used in this study, Protein FDR Confidence: Combined - level of confidence of the identified protein groups (High = q-value < 0.01), negative Log Student's T-test p-value: -Log p-value calculated using the two-sided t-test via Perseus, Student's T-test q-value: q-value calculated using permutation-based FDR via Perseus, Student's Ttest Difference: calculated using the two-sided t-test via Perseus, Student's T-test Test statistic: calculated via Perseus

Supplementary Data 6: Comp. prot. MG08: Proteome Discoverer 3.0 output of Methylocapsa gorgona MG08 proteome data. "Unfiltered proteome data used for comparative proteomics analyses. Protein FDR Confidence: Combined - level of confidence of the identified protein groups, Label - Gene ID used by Microscope platform, Description - protein designation by Microscope platform, Exp. q-value: Combined - displays the q-values derived from the validation, Coverage [%] - percentage of the protein sequences covered by identified peptides, # PSMs - the number of identified peptide spectrum matches identified from all included searches, # Unique Peptides - total number of peptides that are unique to a particular protein, # AAs - length of the protein sequence"

Supplementary Data 7: Comp. prot. SV97: "Unfiltered proteome data used for comparative proteomics analyses. Protein FDR Confidence: Combined - level of confidence of the identified protein groups, Label - Gene ID used by Microscope platform, Description - protein designation by Microscope platform, Exp. q-value: Combined - displays the q-values derived from the validation, Coverage [%] - percentage of the protein sequences covered by identified peptides, # PSMs - the number of identified peptide spectrum matches identified from all included searches, # Unique Peptides - total number of peptides that are unique to a particular protein, # AAs - length of the protein sequence".

Supplementary Data 8: Comp. prot. NE2: Proteome Discoverer 3.0 output of Methylocapsa palsarum NE2 proteome data. "Unfiltered proteome data used for comparative proteomics analyses. Protein FDR Confidence: Combined - level of confidence of the identified protein groups, Label - Gene ID used by Microscope platform, Description - protein designation by Microscope platform, Exp. q-value: Combined - displays the q-values derived from the validation, Coverage [%] - percentage of the protein sequences covered by identified peptides, # PSMs - the number of identified peptide spectrum matches identified from all included searches, # Unique Peptides - total number of peptides that are unique to a particular protein, # AAs - length of the protein sequence".

Supplementary Data 9: Specific affinity: Affinity for CH4, maximal CH4 oxidation rate, and specific affinity for CH4 of Methylocapsa gorgona MG08 and Methylocapsa palsarum NE2 (Fig. 5)

Supplementary Data 10: Putative CODH NE2: Blast-search results of putative CODH subunits encoded in the genome of Methylocapsa palsarum NE2

Supplementary Data 11: Putative CODH SV97: Blast-search results of putative CODH subunits encoded in the genome of Methylocystis rosea SV9

Supplementary Data 12: Top10prot_MG08: Top 10% proteins of Methylocapsa gorgona MG08 contributing most to the inertia of the correspondence analysis (Supplementary Fig. 3). EGGNOG Automatic Classification provided by MicroScope platform. high_1 : atm_4 - normalized, imputed, and log2 fold transformed protein abundances of the replicates (high_ = 1000 p.p.m.v. CH4, atm_ = 1.9 p.p.m.v CH4).

Supplementary Data 13: Top10prot_NE2: Top 10% proteins of Methylocapsa palsarum NE2 contributing most to the inertia of the correspondence analysis (Supplementary Fig. 3). EGGNOG Automatic Classification provided by MicroScope platform. high_1 : atm_4 - normalized, imputed, and log2 fold transformed protein abundances of the replicates (high_ = 1000 p.p.m.v. CH4, atm_ = 1.9 p.p.m.v CH4)

Supplementary Data 14: Top10prot_SV97: Top 10% proteins of Methylocystis rosea SV97 contributing most to the inertia of the correspondence analysis (Supplementary Fig. 3).

Supplementary Data 15: at% 15N enrichment: Cellular 15N – enrichment. OpenMIMS analysis of NanoSIMS images of Methylocapsa gorgona NE2 exposed to 23 at% 15N2. Roi - region of interest.

Supplementary Data 16: at% 15N control: Cellular 15N – control. OpenMIMS analysis of NanoSIMS images of Methylocapsa gorgona MG08 exposed to natural abundant 15N2 in air. Roi - region of interest.
