# Supplementary material for: Physiological basis for atmospheric methane oxidation and methanotrophic growth on air
Source: Nat Commun. 2024 May 16;15:4151. doi: 10.1038/s41467-024-48197-1 (PMC11519548; doi:10.1038/s41467-024-48197-1)
Supplement: Supplementary file 5 — Reporting Summary [file 41467_2024_48197_MOESM5_ESM.pdf]

nature portfolio

Corresponding author(s): Tilman Schmider, Alexander T. Tveit

Last updated by author(s): Mar 26, 2024

Reporting Summary

Nature Portfolio wishes to improve the reproducibility of the work that we publish. This form provides structure for consistency and transparency in reporting. For further information on Nature Portfolio policies, see our Editorial Policies and the Editorial Policy Checklist.

Statistics

For all statistical analyses, confirm that the following items are present in the figure legend, table legend, main text, or Methods section.

n/a	Confirmed	
☐	☒	The exact sample size (n) for each experimental group/condition, given as a discrete number and unit of measurement
☐	☒	A statement on whether measurements were taken from distinct samples or whether the same sample was measured repeatedly
☐	☒	The statistical test(s) used AND whether they are one- or two-sided<br>*Only common tests should be described solely by name; describe more complex techniques in the Methods section.*
☒	☐	A description of all covariates tested
☐	☒	A description of any assumptions or corrections, such as tests of normality and adjustment for multiple comparisons
☐	☒	A full description of the statistical parameters including central tendency (e.g. means) or other basic estimates (e.g. regression coefficient) AND variation (e.g. standard deviation) or associated estimates of uncertainty (e.g. confidence intervals)
☐	☒	For null hypothesis testing, the test statistic (e.g. F, t, r) with confidence intervals, effect sizes, degrees of freedom and P value noted<br>*Give P values as exact values whenever suitable.*
☒	☐	For Bayesian analysis, information on the choice of priors and Markov chain Monte Carlo settings
☒	☐	For hierarchical and complex designs, identification of the appropriate level for tests and full reporting of outcomes
☐	☒	Estimates of effect sizes (e.g. Cohen's d, Pearson's r), indicating how they were calculated

Our web collection on statistics for biologists contains articles on many of the points above.

Software and code

Policy information about availability of computer code

Data collection	GC data were collected using Chromeleon 7.2. Proteome data were collected using Proteome Discvoverer 3.0. Flow cytometry data were collected using BD FACSDiva 8.0.1. Buoyant mass data of cells were collected using the LifeScale built-in software.
Data analysis	The following software was used to process and analyze data: Python 3 version 3.9.13, R version 4.2.2, Chromeleon 7.2, Proteome Discvoverer 3.0, BD FACSDiva 8.0.1, LifeScale built-in software, Python packages: pandas version 1.4.4, seaborn version 0.11.2, matplotlib version 3.5.2, R packages: ggplot2 version 3.2.0, ca<br>version 0.71.1, factoextra version 1.0.7, nlstools version 2.0.0

For manuscripts utilizing custom algorithms or software that are central to the research but not yet described in published literature, software must be made available to editors and reviewers. We strongly encourage code deposition in a community repository (e.g. GitHub). See the Nature Portfolio guidelines for submitting code & software for further information.

Data

Policy information about availability of data

All manuscripts must include a data availability statement. This statement should provide the following information, where applicable:

- Accession codes, unique identifiers, or web links for publicly available datasets
- A description of any restrictions on data availability
- For clinical datasets or third party data, please ensure that the statement adheres to our policy

Proteomics data have been deposited to the ProteomeXchange Consortium via the PRIDE partner repository with the dataset identifier PXD046190 (http://www.ebi.ac.uk/pride/archive/projects/PXD046190). Data used in the study are provided in the supplementary excel files "Source Data" and "Supplementary Data"

Research involving human participants, their data, or biological material

Policy information about studies with human participants or human data. See also policy information about sex, gender (identity/presentation), and sexual orientation and race, ethnicity and racism.

Reporting on sex and gender	*Use the terms sex (biological attribute) and gender (shaped by social and cultural circumstances) carefully in order to avoid confusing both terms. Indicate if findings apply to only one sex or gender; describe whether sex and gender were considered in study design; whether sex and/or gender was determined based on self-reporting or assigned and methods used. Provide in the source data disaggregated sex and gender data, where this information has been collected, and if consent has been obtained for sharing of individual-level data; provide overall numbers in this Reporting Summary. Please state if this information has not been collected. Report sex- and gender-based analyses where performed, justify reasons for lack of sex- and gender-based analysis.*
Reporting on race, ethnicity, or other socially relevant groupings	*Please specify the socially constructed or socially relevant categorization variable(s) used in your manuscript and explain why they were used. Please note that such variables should not be used as proxies for other socially constructed/relevant variables (for example, race or ethnicity should not be used as a proxy for socioeconomic status). Provide clear definitions of the relevant terms used, how they were provided (by the participants/respondents, the researchers, or third parties), and the method(s) used to classify people into the different categories (e.g. self-report, census or administrative data, social media data, etc.) Please provide details about how you controlled for confounding variables in your analyses.*
Population characteristics	*Describe the covariate-relevant population characteristics of the human research participants (e.g. age, genotypic information, past and current diagnosis and treatment categories). If you filled out the behavioural & social sciences study design questions and have nothing to add here, write "See above."*
Recruitment	*Describe how participants were recruited. Outline any potential self-selection bias or other biases that may be present and how these are likely to impact results.*
Ethics oversight	*Identify the organization(s) that approved the study protocol.*

Note that full information on the approval of the study protocol must also be provided in the manuscript.

Field-specific reporting

Please select the one below that is the best fit for your research. If you are not sure, read the appropriate sections before making your selection.

☒ Life sciences ☐ Behavioural & social sciences ☐ Ecological, evolutionary & environmental sciences

For a reference copy of the document with all sections, see nature.com/documents/nr-reporting-summary-flat.pdf

Life sciences study design

All studies must disclose on these points even when the disclosure is negative.

Sample size	In the study, seven methanotrophic strain have been investigated. In each experiment at least 4 replicates per strains were used. Four replicates were sufficient as the findings were highly reproducible.
Data exclusions	All data have been included.
Replication	The trace gas oxidation experiments have been repeated three times. Each replication attempt was successful. The growth with air as sole carbon and energy source experiments were successfully repeated at least 3 times per strain. The growth with air as sole carbon, energy, and nitrogen source experiments were successfully repeated at least 2 times per strain. The 15N incorporation on air experiment has not been repeated.
Randomization	Due to the nature of the experiments, randomization was not required. Covariates control was not relevant as all experiments were carried out under constant conditions

Blinding	Blinding was not applicable to this kind of experiments.

Reporting for specific materials, systems and methods

We require information from authors about some types of materials, experimental systems and methods used in many studies. Here, indicate whether each material, system or method listed is relevant to your study. If you are not sure if a list item applies to your research, read the appropriate section before selecting a response.

Materials & experimental systems

n/a	Involved in the study
☒	☐ Antibodies
☒	☐ Eukaryotic cell lines
☒	☐ Palaeontology and archaeology
☒	☐ Animals and other organisms
☒	☐ Clinical data
☒	☐ Dual use research of concern
☒	☐ Plants

Methods

n/a	Involved in the study
☒	☐ ChIP-seq
☐	☒ Flow cytometry
☒	☐ MRI-based neuroimaging

Plants

Seed stocks	*Report on the source of all seed stocks or other plant material used. If applicable, state the seed stock centre and catalogue number. If plant specimens were collected from the field, describe the collection location, date and sampling procedures.*
Novel plant genotypes	*Describe the methods by which all novel plant genotypes were produced. This includes those generated by transgenic approaches, gene editing, chemical/radiation-based mutagenesis and hybridization. For transgenic lines, describe the transformation method, the number of independent lines analyzed and the generation upon which experiments were performed. For gene-edited lines, describe the editor used, the endogenous sequence targeted for editing, the targeting guide RNA sequence (if applicable) and how the editor was applied.*
Authentication	*Describe any authentication procedures for each seed stock used or novel genotype generated. Describe any experiments used to assess the effect of a mutation and, where applicable, how potential secondary effects (e.g. second site T-DNA insertions, mosiacism, off-target gene editing) were examined.*

Flow Cytometry

Plots

Confirm that:

☒ The axis labels state the marker and fluorochrome used (e.g. CD4-FITC).

☒ The axis scales are clearly visible. Include numbers along axes only for bottom left plot of group (a 'group' is an analysis of identical markers).

☒ All plots are contour plots with outliers or pseudocolor plots.

☒ A numerical value for number of cells or percentage (with statistics) is provided.

Methodology

Sample preparation	The sample preparation is described in the method section of the manuscript. Cells from filter cultures of Methylocapsa gorgona MG08, Methylocapsa palsarum NE2, Methylocystis rosea SV97, and Methylocapsa were prepared as follows: The filters were placed on 150 µL 25x SYBR green I with cells facing upwards and incubated for 10 minutes in dark. Next, the cells were washed twice by letting the filters float on milliQ water for 5 minutes in dark. To remove the cells from the filters, the filters were transferred into 5 mL tubes containing 2 mL 10x diluted solution 1 (DSMZ medium 921), so that the filters sticked to the tube wall and the cells faced inwards. The tubes were vortexed for 10 minutes at medium speed. After vortexing, remaining cells on the filters were washed off and collected by rinsing the filters with 1 mL solution 1 (DSMZ medium 921). The 3 mL cell suspension resulting from the cell removal was spiked with 50 µL absolute counting beads.
Instrument	BD FACSAria III, BD Biosciences, Franklin Lakes, New Jersey, USA
Software	The Software BD FACS Diva Software was used to collect and analyze the flow cytometry data.
Cell population abundance	The samples were not sorted. The flow cytometry was only used to quantify the cell number of the filter cultures.
Gating strategy	The flow cytometer was set up to capture SYBR Green I (excitation = 498 nm, emission = 522 nm) in the green channel and the Absolute Counting Beads (excitation = 350 – 810 nm, emission = 385 – 860 nm) in the blue channel. Unstained cells of M. gorgona MG08, M. palsarum NE2, M. rosea SV97, and M. aurea KYG were used to define the autofluorescence threshold in

the different channels. Size beads (NPPS-4K, Spherotech, Lake Forest, Illinois, USA) were used to draw the forward scatter gate covering events between 0.58 and 4 µm.

☒ Tick this box to confirm that a figure exemplifying the gating strategy is provided in the Supplementary Information.
